# Dyslipidaemia pattern and prevalence among type 2 diabetes mellitus patients on lipid-lowering therapy at a tertiary hospital in central South Africa

**DOI:** 10.1186/s12902-021-00813-7

**Published:** 2021-08-08

**Authors:** Lebohang Pitso, Thabiso Rafaki Petrus Mofokeng, Riette Nel

**Affiliations:** 1 Division of Endocrinology, Department of Internal Medicine, Faculty of Health Sciences, University of the Free State, Universitas Academic Hospital, Bloemfontein, South Africa; 2 Department of Internal Medicine, Faculty of Health Sciences, University of the Free State, 205 Nelson Mandela Drive, 9300 Bloemfontein, South Africa; 3 Department of Internal Medicine, Faculty of Health Sciences, University of the Free State, Universitas Academic Hospital, Bloemfontein, South Africa; 4 Department of Biostatistics, Faculty of Health Sciences, University of the Free State, Bloemfontein, South Africa

**Keywords:** Atherosclerotic cardiovascular disease, ASCVD, Type 2 diabetes mellitus, T2DM, Dyslipidaemia, Lipid-lowering therapy, Low-density lipoprotein cholesterol, LDL-C, Statin

## Abstract

**Background:**

Atherosclerotic cardiovascular disease (ASCVD) is a major cause of death worldwide. A large number of deaths due to ASCVD occurs among people with diabetes mellitus (DM). One of the important modifiable risk factors associated with ASCVD is dyslipidaemia and its prevalence is not known in central South Africa (SA). This study aimed to determine the pattern and prevalence of dyslipidaemia among type 2 diabetes mellitus (T2DM) patients on lipid-lowering therapy.

**Methods:**

This descriptive, retrospective study of patients’ records was conducted at Universitas Academic Hospital in Bloemfontein, SA. The study population included 143 consecutive T2DM patients of any age that attended the Diabetes Clinic from 1 January to 31 March 2019. The patients had to be on lipid-lowering therapy for a minimum duration of 3 months. Data were sourced from the clinic files and included the patient’s lipid profile, anthropometric and demographic data. Dyslipidaemia was defined using the 2018 SA dyslipidaemia guidelines.

**Results:**

The median age of the participants was 63 years (interquartile range [IQR] 52–71 years). The majority of the participants were female (*n* = 92; 64.3 %). The median duration since the DM diagnosis was 18 years (IQR 13–23 years). The prevalence of dyslipidaemia was 86.7 % (*n* = 124). Combined dyslipidaemia, namely either triglycerides (TG) + low-density lipoprotein cholesterol (LDL), high-density lipoprotein cholesterol (HDL) + TG or HDL + LDL, was the most common pattern (*n* = 51; 42.5 %) largely due to raised TG + LDL contributing 37.2 % (*n* = 19) to this pattern. The second and third most common patterns were isolated (either LDL, HDL or TG) and mixed dyslipidaemia (TG + HDL + LDL) at 40.8 % (*n* = 49) and 16.7 % (*n* = 20), respectively. The most frequent lipid abnormality (*n* = 84; 70.0 %) was LDL of ≥ 1.8 mmol/L. Of the 140 participants on statin therapy, only 5 % were on high-intensity therapy.

**Conclusions:**

A high prevalence of dyslipidaemia among DM patients was observed, despite the use of lipid-lowering therapy in this small observational study. Our findings highlight the need to better educate healthcare providers regarding the intensification of lipid-lowering therapy, along with improved strategies to address poor glycaemic control and other modifiable lifestyle factors.

## Background

Diabetes mellitus (DM) is on the increase globally and most alarmingly in the Africa region. An estimated 463 million of the global adult population were living with DM in 2019, and the global prevalence has doubled to 9.3 % since 2000. The Africa region has the highest proportion in the world of both undiagnosed DM (59.7 %) and DM-related deaths occurring under 60 years of age (73.1 %). Atherosclerotic cardiovascular disease (ASCVD) is the largest contributor to both morbidity and mortality for people with DM, with the relative risk of ASCVD between 1.6 and 2.6 [1].

Dyslipidaemia associated with DM is an important modifiable metabolic risk factor to reduce ASCVD [1]. Diabetic dyslipidaemia, also known as atherogenic lipoprotein phenotype (ALP) or atherogenic dyslipidaemia, manifests with elevated fasting and postprandial triglycerides (TG), low high-density lipoprotein cholesterol (HDL-C) and normal to mildly elevated low-density lipoprotein cholesterol (LDL-C) with the predominance of atherogenic small dense low-density lipoprotein (sdLDL) particles [2–4]. This pattern is mainly due to hepatic overproduction of TG-rich very-low-density lipoprotein (VLDL) particles and accelerated exchange of TG in VLDL for cholesteryl esters in HDL and LDL producing sdLDL [2, 3, 5].

Elevated LDL-C, a form of dyslipidaemia, as the cause of ASCVD is unequivocal [6, 7]. Coronary artery disease (CAD), ischaemic stroke and peripheral vascular disease (PVD) are all increased two- to four-fold in the DM population, while heart failure risk is even greater with the risk reported as high as eight-fold in some studies [8–11].

Lowering cholesterol levels, among other metabolic risk factors, can significantly reduce the risk of ASCVD outcomes [1]. Statin therapies have been shown to significantly reduce ASCVD events, including in people with type 2 diabetes mellitus (T2DM) [4, 12, 13]. Among the DM population, cardiovascular (CV) benefits with the use of statins are seen for both primary and secondary prevention [14, 15]. The 5-year incidence of major cardiovascular disease (CVD) events is reduced by 23 % for every 1 mmol/L reduction in LDL-C [4]. As a result, statin therapy is recommended as a first-line treatment for primary and secondary prevention of ASCVD by major society guidelines [4, 16].

Ezetimibe, a non-statin therapy, has been proven to result in additional lowering of LDL-C levels when added to statin therapy and significantly reduced primary composite CV endpoint [17]. Approximately 27 % of this study population had the diagnosis of DM [17].

Both the South African (SA) dyslipidaemia and T2DM guidelines classify T2DM as a high CV risk condition and CV risk scoring is not needed to initiate statin therapy for primary CVD prevention [18, 19]. Based on these guidelines, most T2DM patients’ recommended LDL-C target is < 1.8 mmol/L. Other recommended lipid targets are TG < 1.7 mmol/L, HDL-C of > 1.2 mmol/L in women and > 1.0 mmol/L in men [18, 19].

Despite the higher risk of ASCVD associated with an abnormal lipid profile, dyslipidaemia is undertreated. In one multi-centre study of over 7 000 participants in Europe, only 45 % of the participants with atherogenic dyslipidaemia were on lipid-lowering therapy [20]. In another SA study that evaluated the achievement of LDL-C targets in patients on lipid-lowering therapy in clinical practice, only 41.4 % of patients achieved their LDL-C target [21]. In the European study, approximately 27 % [20] and in the SA study 64 % [21] of the participants had T2DM. A previous report assessing lipid goal attainment in patients on lipid-lowering therapy in central SA was done in the private sector and included patients with and without DM [21].

In our setting, there is a lack of data regarding the prevalence and pattern of dyslipidaemia among T2DM patients on lipid-lowering therapy. Additionally, data regarding the attainability of lipid targets using lipid-lowering therapy are not available. Therefore, this study aimed to determine the pattern and prevalence of dyslipidaemia among T2DM patients on lipid-lowering therapy attending a public sector tertiary Diabetes Clinic in the Free State province of SA. Also, the attainability of the LDL-C treatment target amongst other lipid level targets was investigated.

## Methods

### Study design and setting

A retrospective study was conducted based on the records of T2DM patients that visited Universitas Academic Hospital Diabetes Clinic, Free State Province, from 1 to 2019 to 31 March 2019. The Diabetes Clinic is based in the public sector and provides tertiary care service to a majority of the 2.9 million inhabitants of the province.

### Inclusion and exclusion criteria

Records of all T2DM patients of any age who had been consulted during the relevant period and were on a minimum of 3 months’ lipid-lowering therapy were enrolled in the study. Patients with type 1 DM (T1DM), gestational diabetes or secondary causes of diabetes, and patients not on lipid-lowering therapy for a minimum period of 3 months, were excluded from the study. Patient records with incomplete information were also excluded.

### Population and sampling

The files were sourced based on the clinic’s diary that keeps a record of all the consultation visits. On average, this referral clinic conducts 960 consultations per year and the majority of the patients are seen at least every six months and most have T2DM. Consecutive sampling was used and a total of 257 patient records were screened during the 3-month study period. The clinical data that were obtained from the patient records included age, gender, ethnicity, T2DM duration, presence or absence of ischaemic heart disease, peripheral vascular disease, stroke, hypothyroidism and current smoking status. Hypertension (HT) was recorded and graded according to the 2014 SA hypertension guidelines [22]. The body mass index (BMI) was recorded and classified according to the World Health Organization (WHO) classification [23].

Therapy for T2DM (oral, insulin or a combination thereof) was also recorded. The drug therapies that the patients were taking, which could potentially influence the lipid profile, were noted and included beta-blockers, diuretics, corticosteroids, oestrogens and anti-retroviral therapy. The laboratory data included the fasting lipid profile that contained total cholesterol (TC), TG, HDL-C, and LDL-C. LDL-C measurement was indirect using the Friedewald equation [18]. If TG exceeded 4.5 mmol/L, LDL-C was not calculated by the laboratory as the equation relies on TG values of ≤ 4.5 mmol/L [18]. Lipid-lowering therapy type and dosage were also recorded. Information was recorded on a data collection sheet and transferred to a Microsoft Excel spreadsheet for statistical analyses.

### Definition of dyslipidaemia

Optimal lipid targets for T2DM have been defined in the 2018 dyslipidaemia guidelines by the South African Heart Association (SA Heart) and the Lipid and Atherosclerosis Society of Southern Africa (LASSA) [18], as well as the 2017 Society for Endocrinology, Metabolism and Diabetes of South Africa (SEMDSA) guidelines for the management of T2DM [19].

Using these guidelines [18, 19], dyslipidaemia was defined if one or more of the following were present: TC ≥ 4.5 mmol/L, TG ≥ 1.7 mmol/L, HDL-C ≤ 1.0 mmol in males, ≤ 1.2 mmol/L in females, and LDL-C ≥ 1.8 mmol/L. When a single abnormal lipid parameter (TC, TG, HDL-C or LDL-C) was present, it was classified as isolated dyslipidaemia. When two lipid parameters (elevated TG, low HDL-C or elevated LDL-C) were detected, it was classified as combined dyslipidaemia. When all three lipid parameters were abnormal (elevated TG, low HDL-C and elevated LDL-C), it was classified as mixed dyslipidaemia.

### Statistical analysis

Variables were reported according to the distribution of the sample and were not normally distributed. Descriptive statistics namely frequencies and percentages for categorical data and medians with interquartile ranges (IQR) and percentiles for numerical data were calculated. The prevalence of dyslipidaemia was calculated and described using 95 % confidence for the prevalence.

Associations were calculated between gender and lipid abnormalities (LDL-C, HDL-C and TG) using the Kruskal-Wallis test, and between gender and HT using the Chi-squared or Fisher’s exact test. A two-sided *p*-value of < 0.05 was considered to be statistically significant.

### Ethical considerations

 Ethics approval was obtained from the Health Sciences Research Ethics Committee (HSREC) of the Faculty of Health Sciences, University of the Free State, before the commencement of the study (UFS-HSD2019/0869/2506). Permission to conduct the study was obtained from the Free State Provincial Department of Health and the Head of the Department of Internal Medicine. All methods used in this study were carried out in accordance with relevant guidelines and regulations. The researcher ensured confidentiality by assigning a study number to each file to record clinical data on the datasheet without recording personal information. Because of the retrospective nature of the study, the fact that the information had previously been collected for routine clinical care and no further sample testing nor administration of treatment was conducted, informed consent was waived by the Ethics Committee.

## Results

### Overview of the population included in the study

**Fig. 1:**
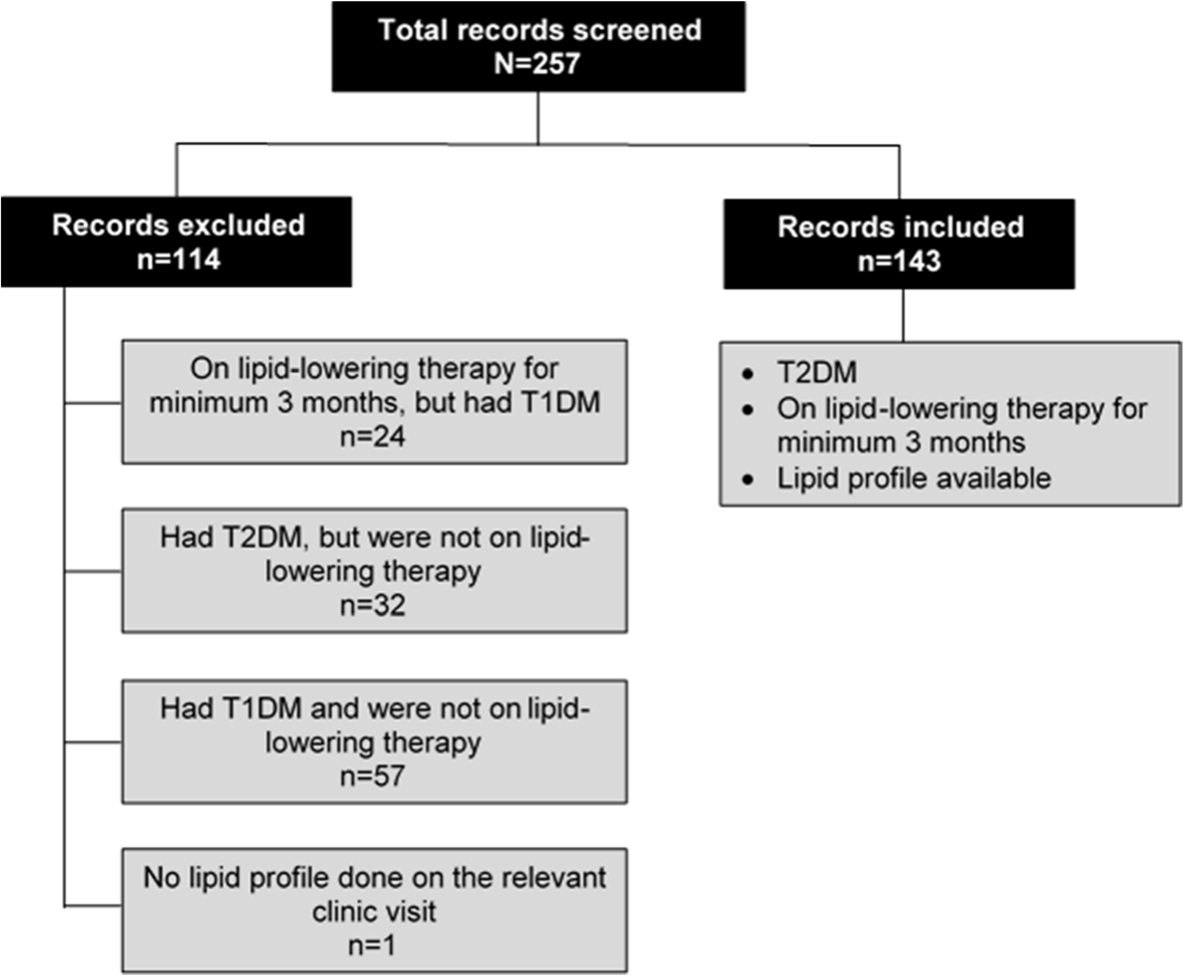
Strobe diagramme summarising patient records screened and the reasons for the inclusion and exclusion of records

Figure 1 summarises the total number of patient records screened and the reasons for the inclusion and exclusion of records. Of the 257 patient records screened over the 3-month study period, 114 were excluded from the study. The remaining143 participants fulfilled the inclusion criteria. All but two participants (*n* = 141; 98.6 %) were classified as very high-risk as per SA dyslipidaemia guidelines, thus requiring LDL-C of < 1.8 mmol/L to be on target. The remaining two (1.4 %) participants’ optimal LDL-C target was < 2.5 mmol/L [18].

### Demographic variables

Most of the participants were aged 40 years or older (*n* = 133; 93 %). The median age of the sample was 63 years (IQR 52–71 years). Most of the participants (*n* = 92; 64.3 %) were female. Slightly more than half of the study participants were of the black ethnic group (*n* = 74; 51.7 %) (Table 1).

**Table 1:** Demographic and clinical information of patients with type 2 diabetes mellitus (*n* = 143, otherwise as indicated)

Variable	n (%)
Age	
Median in years (IQR)	63 (52-71)
Gender	
Male	51 (35.7)
Female	92 (64.3)
Ethnicity	
Black	74 (51.7)
Mixed race	11 (7.7)
White	58 (40.6)
Ischaemic heart disease	30 (21.0)
Stroke	7 (4.9)
Cigarette smoking (*n* = 113)	12 (10.6)
Peripheral vascular disease	9 (6.3)
Hypertension	131 (91.6)
Hypothyroidism	31 (21.7)
Body mass index ≥ 30 kg/m^2^ (*n* = 127)	85 (66.9)
Blood pressure	
Grade 1 hypertension	13 (9.1)
Grade 2 hypertension	8 (5.6)
Grade 3 hypertension	9 (6.3)
Isolated systolic hypertension	45 (31.5)
Antidiabetic therapy	
Oral therapy only	5 (3.5)
Insulin therapy only	53 (37.1)
Dual oral and insulin therapy	85 (59.4)
HbA1c^a^ >7 %	113 (79.0)
Lipid-lowering therapy	
Statin only	135 (94.4)
Fibrate only	3 (2.1)
Dual statin and fibrate therapy	5 (3.5)

^a^*HbA1c* glycated haemoglobin

### Clinical characteristics of the population

The clinical characteristics of the cohort are summarised in Table 1. The median duration of DM diagnosis was 18 years (IQR range 13–23 years). Of the 127 study participants whose anthropometric data were available, two thirds (*n* = 85; 66.9 %) were obese with a median BMI of 34.1 kg/m^2^ (IQR 28.4–38.9 kg/m^2^). Female participants had a higher median BMI than male participants (35.6 versus 31.6 kg/m^2^; Kruskal-Wallis test, Chi-squared (*x*^2^) = 4.67, *p* = 0.03). Glycated haemoglobin (HbA1c) > 7 %, implying poor control, was noted in 113 (79.0 %) patients, with a median HbA1c of 8.9 % (IQR 7.4–10.1 %). A diagnosis of hypothyroidism was recorded in 21.7 % (*n* = 31/142) of participants, with a median thyroid-stimulating hormone (TSH) level of 2.4 mIU/L (IQR 1.6–3.6 mIU/L), demonstrating good control of hypothyroidism.

Nearly two-thirds of the sample (*n* = 92; 64.3 %) and slightly less than one third (*n* = 45; 31.5 %), respectively, were on a diuretic and beta-blocker therapy (Table 2). Approximately a quarter of the patients (*n* = 37; 25.9 %) were on concomitant diuretic and beta-blocker therapy in keeping with the high rate of hypertension (*n* = 131; 91.6 %) observed among the cohort (Table 1). The prevalence of hypertension in males was similar to that in females (90.2 vs. 92.4 %; Fisher’s exact test *p* = 0.7). Among four patients on human immunodeficiency virus antiretroviral therapy (HIV ART) (Table 2), two were documented to be on efavirenz-based therapy, while in the remaining two, antiretroviral therapy regimen information could not be retrieved from the file records.

**Table 2:** Patient drug therapy list (*n* = 143) that may influence lipid profiles

Type of agent	n (%)
Corticosteroids	2 (1.4)
Diuretics	94 (64.3)
Oestrogens	2 (1.4)
Beta-blockers	45 (31.5)
HIV antiretroviral therapy (ART)	4 (2.8)

### Lipid-lowering therapy

The most common lipid-lowering therapy among the cohort was a statin monotherapy (*n* = 135; 94.4 %), followed by dual statin and fibrate (*n* = 5; 3.5 %) and a fibrate monotherapy (*n* = 3; 2.1 %) (Table 1). No patients were on any other type of lipid-lowering therapy including ezetimibe. The most commonly prescribed statin therapy was simvastatin (*n* = 128; 91.4 %) followed by atorvastatin (*n* = 12; 8.6 %). No patients were on rosuvastatin or any other type of statin. The median dose of simvastatin in 128 participants was 30 mg (IQR 20–40 mg), while in 12 participants on atorvastatin, the median dose was also 30 mg (IQR 20–40 mg). As shown in Table 3, of the 140 patients on a statin with or without a fibrate, only seven (5.0 %) were on a high-intensity statin.

**Table 3:** Classification of statins by potency of LDL-C lowering (*n* = 140)

Potency	n (%)
High-intensity statin	
Atorvastatin 40 mg	6 (4.3)
Simvastatin 80 mg	1 (0.7)
Moderate-intensity statin	
Atorvastatin 10 mg	2 (1.4)
Atorvastatin 20 mg	4 (2.9)
Simvastatin 20 mg	57 (40.7)
Simvastatin 40 mg	63 (45.0)
Low-intensity statin	
Simvastatin 10 mg	7 (5.0)

### Dyslipidaemia pattern and prevalence

**Fig. 2:**
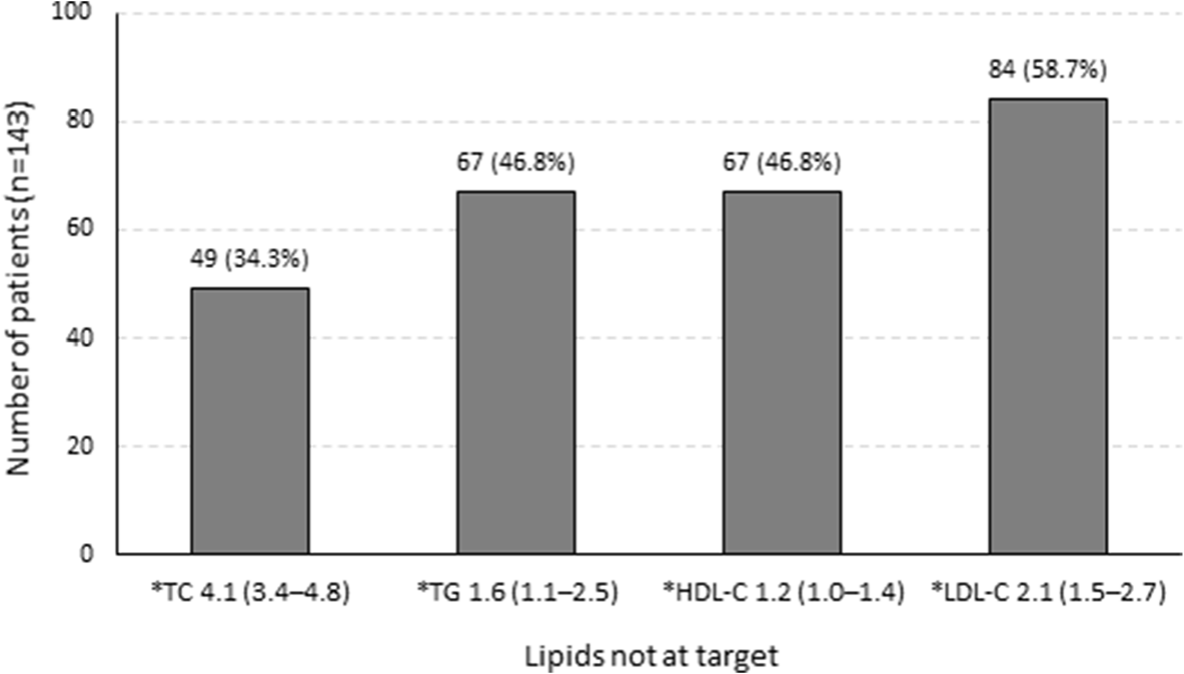
Individual lipid abnormalities and the median levels of the lipid profile among the study participants (*n* = 143). *Median values in mmol/L with interquartile ranges in brackets. TC: total cholesterol ≥ 4.5 mmol/L; TG: triglycerides ≥ 1.7 mmol/L; HDL-C: high-density lipoprotein cholesterol ≥ 1.0 mmol/L (male) and ≥ 1.2 mmol/L (female); LDL-C: low-density lipoprotein cholesterol ≥ 1.8 mmol/L

The distribution of the pattern of dyslipidaemia is shown in Table 4. The prevalence of dyslipidaemia among the cohort was 86.7 % (*n* = 124/143) (95 % confidence interval for the prevalence [80.2 %; 91.3 %]). The most frequent lipid abnormality was high LDL-C (*n* = 84/143) in 54.9 and 60.9 % of the male and female patients, respectively (Fig. 2).

**Table 4:** Distribution of the pattern of dyslipidaemia among the study cohort (*n* = 143)

Lipids outside target	Parameter	n (%)	Total
No dyslipidaemia	TG/HDL/LDL	19 (13.3)	19 (13.3)
Isolated dyslipidaemia	TG	7 (4.9)	49 (34.3)
	HDL	12 (8.4)	
	LDL	30 (21.0)	
Combined dyslipidaemia	TG + LDL	19 (13.3)	51 (35.7)
	TG + HDL	17 (11.9)	
	HDL + LDL	15 (10.5)	
Mixed dyslipidaemia	TG + HDL + LDL	20 (14.0)	20 (14.0)
Unclassified pattern	LDL not calculated	4 (2.8)	4 (2.8)

*TG* triglycerides, *HDL* high-density lipoprotein, *LDL* low-density lipoprotein

The finding of 84 participants with elevated LDL-C meant that in 58.7 % of the cohort (70 % of patients with dyslipidaemia and full lipid profile), LDL-C was not on target (Fig. 2). The median lipid profile indices of the cohort are illustrated in Fig. 2 and the comparison between males and females is shown in Table 5. Female patients had a significantly higher median LDL-C (2.2 mmol/L versus 1.9 mmol/L; Kruskal-Wallis test, *x*^2^ = 4.38, *p* = 0.04) (Table 5). The second most common lipid abnormality was both high TG and low HDL-C (*n* = 67 each), affecting 46.8 % of the patient cohort (Fig. 2). For elevated TG, 37.2 % of males and 52.2 % of females contributed to the prevalence with a similar median TG level (1.5 mmol/L versus 1.8 mmol/L; Kruskal-Wallis test, *x*^2^ = 2.30, *p* = 0.1). In 41.2 % of males and 50.0 % of females, HDL-C was below target, with the median HDL-C significantly lower in males compared to females (1.1 mmol/L versus 1.2 mmol/L; Kruskal-Wallis test, *x*^2^ = 5.44, *p* = 0.02) (Table 5).

**Table 5:** Comparison of the lipid profile of male and female patients

Lipid component	Total (*n* = 143)^a^	Male (M) (*n* = 51)	Female (F) (*n* = 92)	*p*-value
TG ≥ 1.7 mmol/L	1.61 (1.15–2.49)	1.55 (1.08–2.07)	1.84 (1.17–2.76)	0.1
HDL-C				
≤ 1.0 mmol/L (M)	1.17 (0.97–1.39)	1.09 (0.87–1.33)	1.21 (1.00–1.43)	0.02
≤ 1.2 mmol/L (F)				
^a^LDL-C ≥ 1.8 mmol/L	2.12 (1.48–2.67)	1.87 (1.27–2.23)	2.22 (1.52–2.77)	0.04

^a^For LDL-C, the total was *n* = 139, M (*n* = 50) & F (*n* = 89)

Only 19 (13.3 %) patients in the study (15.7 % of males and 11.9 % of females) had all four lipid parameters at target (Table 4). The most common dyslipidaemia pattern among patients with dyslipidaemia and full lipid indices (*n* = 51/120) was combined dyslipidaemia at 42.5 %, representing 35.7 % of all the patients in the study (Table 4). It was followed by isolated (*n* = 49) and mixed (*n* = 20) dyslipidaemia patterns at 40.8 % (34.3 % of the cohort) and 16.7 % (14 % of the cohort), respectively. High LDL-C plus either high TG or low HDL-C was common in males (32.6 % of males), whereas in females, high TG plus either low HDL-C or high LDL-C was most prevalent (29.6 % of females) (Table 6).

**Table 6:** Dyslipidaemia pattern among male and female patients with diabetic dyslipidaemia

Pattern of dyslipidaemia	Male (*n* = 43)	Female (*n* = 81)	Total (*n* = 124)
	**n (%)**	**n (%)**	**n (%)**
Isolated	20 (46.5)	29 (35.8)	49 (39.5)
High TG	3 (7.0)	4 (4.9)	7 (5.6)
Low HDL-C	6 (13.9)	6 (7.4)	12 (9.7)
High LDL-C	11 (25.6)	19 (23.5)	30 (24.2)
Combined	19 (44.2)	32 (39.5)	51 (41.1)
High TG + low HDL-C	5 (11.6)	12 (14.8)	17 (13.7)
High TG + high LDL-C	7 (16.3)	12 (14.8)	19 (15.3)
Low HDL + high LDL-C	7 (16.3)	8 (9.9)	15 (12.1)
Mixed (high TG + low HDL-C + high LDL-C)	3 (7.0)	17 (21.0)	20 (16.1)
Unclassified	1 (2.3)	3 (3.7)	4 (3.2)

*TG* triglycerides, *HDL* high-density lipoprotein cholesterol, *LDL-C* low-density lipoprotein cholesterol

In approximately 3 % of the patients (2.8 % of the total cohort or 3.2 % of patients with dyslipidaemia), the pattern of dyslipidaemia could not be classified (Tables 4 and 6). This was due to LDL-C that had not been calculated because TG was > 4.5 mmol/L, making the Friedewald equation estimation of LDL-C not reliable.

## Discussion

In this study of T2DM patients at high risk for cardiovascular disease, we observed that 58.7 % of the patients were not achieving the LDL-C target of < 1.8 mmol/L as recommended by SA guidelines. We found a very high prevalence of dyslipidaemia (86.7 % in our study) despite the use of lipid-lowering therapy. The most common lipid pattern abnormality among these patients was combined dyslipidaemia attributable largely to TG and LDL-C above target.

Dyslipidaemia prevalence remains high in our study population despite the use of lipid-lowering therapy. Given that hypercholesterolaemia caused 4.4 million deaths as reported in the 2016 Global Burden of Disease study [24], 86.7 % of patients in our study remain at high risk for mortality. Elsewhere in South Africa similar prevalence has been reported, ranging between 87.5 and 93.5 % in T2DM patients [25, 26]. It is worth noting that even though the two SA studies had been conducted in the public sector, the use of lipid-lowering therapy magnitude was different from our study. In the Naidoo study [26], 83 % of the participants were on any lipid-lowering therapy, and all the patients in the Daya study [25] were only on simvastatin. In our study in comparison, all patients were on lipid-lowering therapy consisting of simvastatin, atorvastatin or bezafibrate. The observation may account for lower dyslipidaemia prevalence in our study. The high prevalence of dyslipidaemia among DM patients on lipid-lowering therapy is not unique to SA and has been observed in international studies with varying prevalence. One large retrospective study in the United Kingdom reported a dyslipidaemia prevalence of 77.1 % in the DM population [27], while in China there was a 70.9 % overall prevalence [28].

Of note is that over half of the patients with T2DM in our study had LDL-C above target and remain at risk of major cardiovascular events despite the use of statin therapy. This finding is similar to an observational study [21] that examined the management of LDL-C levels in SA, reporting that 58.6 % of the patients on lipid-lowering therapy did not reach the LDL-C target. Similar to our study, 98.7 % (97.9 % in our study) of participants were on statin therapy in this study [21]. In contrast, however, approximately two-thirds of patients in this study (65.2 %) had DM compared to 100 % in our study and 57.9 % were patients managed in the private sector compared to 100 % in the public sector in our study [21]. When focusing on the under-resourced South African public healthcare setting only, as is the case with our study, two other SA studies have found an even higher proportion of patients not meeting target LDL-C of < 1.8 mol/L ranging from 73.5 to 76.5 % [25, 26]. Of note, in the Daya study, only simvastatin was used by all patients at a mean dose of 20 mg [25], whereas in our study 128 out of 140 patients on statin therapy (89.5 % of the patients) used simvastatin at the higher median dose of 30 mg. The remaining 10.5 % of patients in our study were on fibrates (2.1 %) and higher potency atorvastatin (8.4 %). The use of the higher median dose of simvastatin and high-potency atorvastatin may account for the higher proportion of patients achieving LDL-C of < 1.8 mmol/L in our study. In the Naidoo study [26], 83 % of patients with DM (compared to 100 % in our study) were on any lipid-lowering therapy. Again, there was a higher percentage of patients on lipid-lowering therapy in our study and that may explain the higher proportion of patients reaching the target LDL-C compared to the Naidoo study. Nonetheless, over 50 % of patients in our study are still undertreated despite 97.9 % of them being on statin therapy.

Globally, the success rate for LDL-C goal attainment differs from the 41.3 % in our study and has ranged from 39.9 to 61.5 % with the highest success rate among the lowest CV risk groups [28, 29]. Numerous high-quality studies have proven elevated LDL-C as a cause of ASCVD [6, 7] and the risk of CAD, stroke and heart failure is increased at least four-fold in DM [8]. Patients in our study remain at high risk for adverse CV events and would benefit from the lowering of LDL-C. The observation of the above target LDL-C and overall high prevalence of dyslipidaemia in our study highlights the lack of optimal management of dyslipidaemia despite the high use of statin therapy.

Although we did not test the reasons behind this poor management of dyslipidaemia in our study, we found that various possibilities are contributing to our findings. We noted in our study that only 5 % of patients receiving statins were on a high-intensity statin. This is surprisingly low given the high dyslipidaemia prevalence and lack of achievement of LDL-C targets. This may be due to clinician inertia and lack of awareness regarding the appropriate use of statin potency and dose titration. Lack of physician awareness of treatment guidelines, underestimation of the patient’s CV risk and clinician apathy to titrate statins have been observed before [28]. In SA, the use of high-intensity statin use has ranged from 8.8 to 25.1 %, with improved use observed in the private sector clinical practice compared to the public sector [21, 26].

Access to high-intensity statins remains a challenge in our low-resource setting. At the time of our study, high-intensity statins such as atorvastatin and rosuvastatin were available only on a motivational basis. Additionally, the supply of the high-intensity statins at the primary care level remains inadequate despite successful motivation and approval for use in selected individual patients. Cholesterol absorption inhibitor was not used in any of the patients despite a high prevalence of dyslipidaemia in our study, particularly the LDL-C above target. This is comparable to other local studies with ezetimibe only used in one (2.6 % of all participants in that study) out of the three studies of patients with a high prevalence of dyslipidaemia [21, 25, 26]. Ezetimibe was shown to have additional LDL-C lowering when added to statin and improved cardiovascular outcomes [17]. Ezetimibe is accessible only at a tertiary care level on an individual motivated basis. Lack of access is also likely contributing to poor use of high-intensity statin and non-use of ezetimibe in our study despite the poor achievement of LDL-C target.

We found a high rate of obesity and this may contribute to the high prevalence of dyslipidaemia, particularly the combined dyslipidaemia pattern that was most prevalent in our study. 66.9 % of patients in our study had BMI ≥ 30 kg/m^2^ and combined dyslipidaemia (mainly high TG plus LDL) was the commonest pattern of dyslipidaemia. Obesity is associated with insulin resistance in T2DM leading to hypertriglyceridaemia, low HDL-C and high LDL-C, particularly sdLDL-C [5].

We also noted in our study that there was poor T2DM control with a median HbA1c of 8.9 % likely contributing to the lipid abnormalities observed. Poor T2DM control has been shown to correlate positively with unfavourable lipid profile [30].

We did not test poor treatment adherence by the patients as a contributor, but lack of adherence is known as a contributor to the underachievement of LDL-C target [28] and may have contributed to our study findings.

Regardless of the reason for patients failing to meet the LDL-C target, it is clear that the residual diabetic dyslipidaemia is associated with high cardiovascular morbidity and mortality [31, 32]. Patients with T2DM would benefit greatly from the appropriate use of lipid-lowering therapy, particularly statins [32].

Combined dyslipidaemia was the most common pattern of dyslipidaemia observed, accounting for 42.5 % of patients with dyslipidaemia and full lipid profiles. This is similar to findings observed elsewhere in South Africa. Daya et al. observed that among T2DM patients with dyslipidaemia, 43.8 % had combined dyslipidaemia that was the most common pattern observed [25]. In our study, the combined dyslipidaemia pattern was driven largely by LDL-C and TG levels not at target in 37.2 % of patients with the combined pattern. This likely reflects residual atherogenic diabetic dyslipidaemia that is not fully treated by pharmacological and non-pharmacological interventions. It has been previously documented that overproduction of hepatic TG-rich VLDL and decreased degradation of apolipoprotein B (apoB), a major component of VLDL, in insulin-deficient and/or resistant individuals contribute significantly to hypertriglyceridaemia [5]. Additionally, elevated LDL largely sdLDL, and glycated LDL that participate in atherosclerosis are observed in individuals with T2DM, obesity and insulin resistance. This atherogenic LDL is the hallmark of diabetic dyslipidaemia [5]. Indeed, patients in our study had high rates of obesity (median BMI 34.1 kg/m^2^) and poorly controlled T2DM (HbA1c > 7 % in 79 % of patients) that could explain the observed dyslipidaemia pattern.

Our study had several limitations that should be noted. As a retrospective study, selection or information bias was inherent and cross-sectional design precluded any temporal association between baseline and subsequent LDL-C levels following lipid-lowering therapy. Dyslipidaemia due to secondary drug causes, such as thiazide diuretics and beta-blockers, could not completely be ruled out as a confounder. We did not collect data on lipid-lowering therapy contraindications, adverse effects or adherence to treatment that might affect the use of lipid-lowering therapy. We also did not collect data on how long the patients were taking lipid-lowering medication for and that could have affected lipid profiles. Because it was conducted in a tertiary diabetes clinic, the study might represent a particular population that is at the highest CV risk and with difficult to control lipid profiles. As a result, the findings may not be generalisable to the entire South African T2DM population. Further large prospective studies reflecting prevalence in secondary care and district hospitals, as well as primary health care clinics in a public sector setting, are recommended to determine the pattern and prevalence of dyslipidaemia. These studies should also assess the availability, access and use of lipid-lowering therapy among the South African T2DM population.

## Conclusions

Our study draws special attention to the underachievement of lipid targets in patients with T2DM who are already taking lipid-lowering therapy. A high prevalence of dyslipidaemia and the underuse of high-intensity statin therapy was observed, despite available clinical guidelines. We highlight the need for better education of both healthcare providers at all levels of care and patients regarding the intensification of lipid-lowering therapy among T2DM whenever appropriate and indicated. Our study also should inform the healthcare policymakers to address the accessibility of the more potent lipid-lowering therapy, such as high potency-statins and additional lipid-lowering therapy like ezetimibe, in the public sector healthcare setting to improve the clinical management of diabetic dyslipidaemia. These measures, along with improved strategies to address poor glycaemic control and modifiable lifestyle factors such as the high rate of obesity and hypertension, could help to reduce excess ASCVD morbidity and mortality associated with residual diabetic dyslipidaemia. A large prospective study in the public sector setting is needed to further evaluate the prevalence and pattern of dyslipidaemia, and the appropriate use and availability of lipid-lowering therapy among T2DM patients.

## Data Availability

The datasets used and/or analysed during the current study are available from the corresponding author on reasonable request.

## Abbreviations

apoB: Apolipoprotein B
BMI: Body mass index
CAD: Coronary artery disease
ASCVD: Atherosclerotic cardiovascular disease
CV: Cardiovascular
DM: Diabetes mellitus
HbA1c: Glycated haemoglobin
HDL-C: High-density lipoprotein cholesterol
HT: Hypertension
LDL-C: Low-density lipoprotein cholesterol
SA: South Africa
sdLDL: Small dense low-density lipoprotein cholesterol
T2DM: Type 2 diabetes mellitus
TG: Triglyceride
TSH: Thyroid-stimulating hormone
VLDL: Very low-density lipoprotein
